# Natal Origin and Spatiotemporal Distribution of Leatherback Turtle (*Dermochelys coriacea*) Strandings at a Foraging Hotspot in Temperate Waters of the Southwest Atlantic Ocean

**DOI:** 10.3390/ani13081285

**Published:** 2023-04-08

**Authors:** Gabriela M. Vélez-Rubio, Laura Prosdocimi, Milagros López-Mendilaharsu, Maria Noel Caraccio, Alejandro Fallabrino, Erin L. LaCasella, Peter H. Dutton

**Affiliations:** 1 Karumbé NGO, Av. Rivera 3245, Montevideo 11600, Uruguay; 2 Sección Oceanografía y Ecología Marina, Instituto de Ecología y Ciencias Ambientales, Facultad de Ciencias, Universidad de la República, Montevideo 11400, Uruguay; 3 Departamento MEDIA, Centro Universitario Regional del Este (CURE), Universidad de la República, Ruta 9 Intersección con Ruta 15, Rocha 27000, Uruguay; 4 Laboratorio de Ecología, Comportamiento y Mamíferos Marinos (LECyMM), Museo Argentino de Ciencias Naturales (MACN-CONICET), Av. Ángel Gallardo 470, CABA, Buenos Aires C1405DJR, Argentina; 5 NOAA-National Marine Fisheries Service, Southwest Fisheries Science Center, 8901 La Jolla Shores Dr, La Jolla, CA 92037, USA

**Keywords:** marine turtles, threats, genetic diversity, Río de la Plata estuary, Uruguay, conservation management and practice

## Abstract

**Simple Summary:**

A better understanding of the geographic distribution of juvenile and adult stages of highly migratory marine reptiles with complex life histories, such as leatherback turtles, is needed in order to develop conservation measures to mitigate threats, both at nesting beaches and distant foraging areas in the Southwest Atlantic Ocean. Our assessment of an important leatherback foraging area in Uruguayan waters over a 25-year period found that the population was composed of large juveniles and adults originating from the genetic breeding populations in West Africa and that the spatiotemporal variability in distribution and abundance of strandings is probably associated with the availability of food resources and patterns of fishing activity. Our results reinforce the need to identify regional habitat use areas across the broader Southwest Atlantic Region.

**Abstract:**

Leatherback turtles migrate long distances between nesting beaches and distant foraging areas worldwide. This study analyzes the genetic diversity, life history stage, spatiotemporal distribution, and associated threats of a foraging aggregation in the Southwest Atlantic Ocean. A total of 242 leatherbacks stranded or bycaught by artisanal fisheries were recorded from 1997 to 2021 in Uruguay, with sizes ranging from 110.0 to 170.0 cm carapace lengths, indicating that the aggregation is composed of large juveniles and adults. Results of Bayesian mixed-stock analysis show that leatherbacks come primarily from the West African rookeries, based on mitochondrial DNA sequences obtained from 59 of the turtles representing seven haplotypes, including a novel one (Dc1.7). The main threat identified in the area is the fisheries bycatch but most of the carcasses observed were badly decomposed. There was significant seasonal and interannual variability in strandings that is likely associated with the availability of prey and the intensity of the fishing effort. Taken together, these findings reinforce the importance of these South American foraging areas for leatherbacks and the need to determine regional habitat use and migratory routes across the broader Atlantic region, in order to develop effective conservation measures to mitigate threats both at nesting beaches and foraging areas.

## 1. Introduction

The leatherback turtle, *Dermochelys coriacea*, is a cosmopolitan migratory species, which breeds in tropical and subtropical regions and is found at widely dispersed foraging areas, occurring throughout temperate oceans of the world [1,2,3]. A total of seven broad subpopulations [4], or Regional Management Units (RMUs) [5] are recognized globally, including the Southwest Atlantic subpopulation, which is listed as Critically Endangered [6,7]. Each of the subpopulations or RMUs encompass demographically distinct nesting populations, referred to as Management Units (MUs), or sometimes genetic stocks, that can include one or more nesting sites (rookeries) [8]. Threats in the South Atlantic Ocean include incidental capture in commercial and artisanal fisheries, boat strikes, debris ingestion, directed harvest, and habitat loss [9,10,11,12,13,14,15,16].

Nesting in the Southwest Atlantic Ocean (SWAO) is relatively scarce and occurs primarily to the north of Espiritu Santo State in Brazil [17], with sporadic nesting in the Brazilian states of Bahia, Rio de Janeiro, and further south in Santa Catarina, and Rio Grande do Sul [18,19,20]. The SWAO nesting assemblage, or MU, is extremely small, with the annual number of reported nests increasing slightly from 26 nests in 1988–1992 to 90 nests in 2013–2017 [21].

Leatherback turtles are obligate predators of gelatinous zooplankton, and they are associated with coastal aggregations of different jellyfish species [22], often using coastal ecosystems, such as estuaries or river plumes as foraging areas [22,23,24,25]. Their diving behavior and habitat use are influenced by seasonal variations in local prey availability [3,26,27,28]. Ephemeral oceanic foraging areas are also patchily distributed in association with mesoscale oceanographic features, such as frontal zones [25,29,30], causing this species to travel over large regions in search of food [27,31].

**Figure 1:**
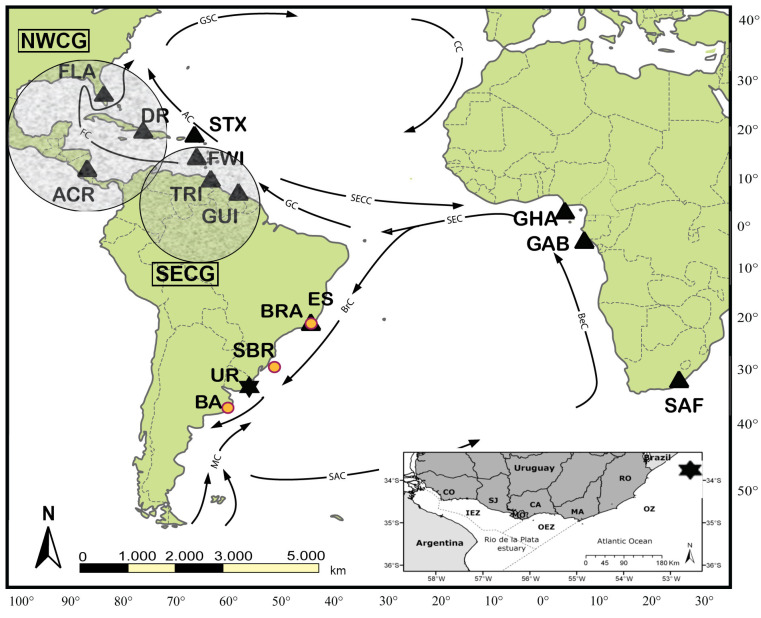
Map indicating the location of the Uruguay (UR) foraging ground (black star) relative to the main Atlantic leatherback nesting colonies (black triangles); BRA (Espirito Santo, Brazil); TRI (Trinidad), GUI (French Guiana and Suriname) and FWI (French West Indies including, Guadaloupe and Martinique), which represent the SECG group; FLA (Florida), DR (Dominican Republic), and ACR (Costa Rica), which represent the NWCG group; STX (St. Croix), GHA (Ghana), GAB (Gabon), and SAF (South Africa). Additional foraging ground sites (yellow circle): Espírito Santo State (ES, Brazil), SBR (Southern Brazil), and BA (Buenos Aires, Argentina) are indicated (yellow circles). Arrows represent oceanic currents: Gulf Stream (GSC), Antilles (AC), Florida (FC), South Equatorial Current (SEC), South Equatorial Counter Current (SECC), Guiana Current (GC), Brazil Current (BrC), Malvinas Current (MC), South Atlantic Current (SAC), and Benguela Current (BeC). The inset shows the Uruguayan coast. The coastal waters could be divided into three regions: Inner Estuarine Zone (IEZ), Outer Estuarine Zone (OEZ), and Oceanic Zone (OZ), which are shown as well as the Departments (CO: Colonia, SJ: San José, MO: Montevideo, CA: Canelones, MA: Maldonado; RO: Rocha).

Although historic reports have indicated the presence of leatherbacks in the SWAO since 1900 [32], it is only in recent decades that substantial foraging aggregations have been identified in the coastal waters off South America, in Brazil [33,34], and in Argentina [35]. Genetic studies using mitochondrial DNA (mtDNA) sequencing and mixed stock analysis (MSA) found that leatherbacks foraging off the coast of Brazil, and along the coast of Argentina were predominantly from populations nesting in West Africa [33,35]. Satellite tracking studies have shown post-nesting migration of leatherbacks from West Africa to waters off South America [3,36,37], and oceanographic dispersal modeling indicated westward dispersal of post-hatchlings from Gabon towards the South American continent (Figure 1) [38].

The southern distribution limit for leatherbacks in the SWAO corresponds to the south of the Buenos Aires province (38°55′ S), based on information from fishery surveys, beach surveys, public reports, and satellite tracking [3,11]. The coastal and oceanic waters in this region are used by large juveniles and adults based on strandings, satellite telemetry studies, and incidental fisheries bycatch information [3,36,39,40,41,42,43,44]. In a recent study based on incidental capture of juvenile leatherbacks in Brazilian and Atlantic international waters, López-Mendilaharsu et al. [38] found the largest juveniles in tropical and subtropical waters (ca. 5° N to 25° S), while small individuals (<80 cm CCL) were restricted to the equatorial central Atlantic, between latitudes 3.5° N and 3.1° S.

There is a paucity of information on leatherbacks present in the territorial waters of Uruguay, despite the recognition of the endangered conservation status of leatherbacks in Uruguay [45,46]. In order to better understand the threats to this highly migratory species beyond the nesting beaches, there is a need to obtain more information on the immature life history stages, and from the data-poor foraging areas of the Southwest Atlantic, as highlighted in a recent review published by Wildermann et al. [47].

Here, we present information obtained from strandings and incidentally caught leatherback turtles in Uruguayan coastal waters, along with the results of the genetic analysis of samples collected. From this information, we determined genetic diversity and natal origin, identified the life history stage, determined the spatiotemporal distribution of the strandings, and identified associated threats to improve our knowledge of Southwest Atlantic foraging aggregations of this species.

## 2. Materials and Methods

### 2.1. Study Area

The Uruguayan coast is part of a complex hydrological system comprising the frontal zone of the Rio de la Plata (RP) estuary and the Atlantic Ocean (Figure 1), primarily influenced by the Malvinas current during the austral Winter and the Brazilian current during the austral Summer [48,49]. This causes variations of >15 °C in sea surface temperatures (range 10–27 °C) throughout the year [50]. The study area includes the entire Uruguayan coast, a total of 710 km (km), from Nueva Palmira (33°53′ S, 58°25′ W), well inside the Uruguay River, to Barra del Chuy (33°44′ S, 53°22′ W), which forms a natural border with Brazil along the Atlantic coast. We divided the study area into three zones based on the differences in hydrological characteristics: an inner estuarine zone (ca. 350 km), from Nueva Palmira to Montevideo, characterized by a fluvial-marine salinity regime (salinity 12) influenced mainly by the RP discharge; an outer estuarine zone (ca. 130 km) from Montevideo to Punta del Este, representing a transition between oceanic and estuarine characteristics with an intermediate salinity range (15–25); and an oceanic zone (ca. 230 km) from Punta del Este to Barra del Chuy, which presents a remarkably oceanic regime (salinity 26) (Figure 1).

### 2.2. Sample Collection

Tissue samples for genetic analysis were collected from turtles encountered as bycatch in artisanal fisheries, or as strandings, beginning in 2002. Skin and muscle samples were collected and preserved in 90% ETOH, following the methodology described by Dutton [51]. Care was taken to use clean materials, in order to avoid cross-contamination during sampling. Tissue samples were shipped to the Southwest Fisheries Science Center in La Jolla, CA, (USA) for processing and were stored in the Marine Mammal and Sea Turtle Research (MMASTR) Collection at −20 °C for long-term storage. Curved carapace length (CCL), from the nuchal notch to the back tip of the caudal peduncle for leatherbacks, was measured for all individuals, as described by Eckert et al. [52].

### 2.3. Laboratory Analysis

Genomic DNA was extracted from each tissue sample using a sodium chloride extraction protocol (modified from Miller et al., 1988) and the mtDNA was amplified and sequenced following laboratory procedures, described in LaCasella et al. [53]. Briefly, a ~800 base pair (bp) portion of the mtDNA control region was amplified using polymerase chain reaction (PCR) methodologies, with primers LCM15382 (5′ GCTTAACCCTAAAGCATTGG 3′), and H950 g (5′ GTCTCGGATTTAGGGGTTTG 3′) (Abreu-Grobois et al., 2006), in a 25 μL PCR reaction, containing 18.25 μL purified H_2_O, 2.5 μL of 10× Mg buffer, 1.5 μL DNTPs, 0.75 μL of each primer, 0.25 μL of Taq polymerase, and 1 μL of template DNA. MJ Research PTC-100 thermocyclers were used for the PCR, with the following profile: initial DNA denaturation at 90 °C for 2 min, followed by 30 cycles of (1) DNA denaturation at 94 °C for 50 s, (2) primer annealing at 56 °C for 50 s, and (3) primer extension at 72 °C for 1 min, and a final primer extension at 72 °C for 5 min. Negative PCR controls were included to monitor for contamination. PCR products were purified by combining 5 μL of product with 2 μL of an Exonuclease I and Shrimp Alkaline Phosphatase solution. Cycle sequencing reactions were conducted in both directions with a Big Dye v3.1 Cycle Sequencing Kit (Applied Biosystems^®^ (ABI) Foster City, CA, USA) and the fragments were analyzed using Sanger sequencing on an ABI 3730 automated genetic analyzer. Sequences from both forward and reverse strands were aligned and edited and individual haplotypes were identified by comparing each sample against a 763 bp reference library, representing known leatherback haplotypes identified in the Atlantic and Pacific [54], using Geneious v6.0.2–8.1.9 [55].

### 2.4. Sexual Maturity, State of Decomposition, and Cause of Stranding Determination

Since marine turtles from multiple populations are found in foraging grounds [35,56,57,58,59,60,61] and marine turtles from different populations may reach sexual maturity at different sizes, the minimum size of leatherback nesting females from the closest nesting colonies in the SWAO [17] was used to categorize the life stage of turtles into two broad categories. Individuals with a much smaller size than 130 cm CCL were considered as small juveniles and individuals ≥to 130 cm CCL were combined into a single large juvenile and adult category. Where possible, sex was determined by external examination of the genitalia and examination of gonads from dead necropsied turtles. The state of decomposition (alive, freshly dead, moderate decomposition, or advanced decomposition), the main cause of death, and any noticeable interaction were also recorded.

To determine the main cause of death, the guidelines proposed by Monteiro et al. [42] were used to identify types of fisheries interactions, where possible. For entanglement in gill nets, evidence such as pieces of monofilament netting around the head, flippers, or carapace was recorded. For longline interactions, the presence of hooks in any part of the body (beak, flippers, among others) was used. Signs of trawl fisheries are difficult to identify because, commonly, fishers use ropes to return heavy turtles brought onboard to the sea. Animals with pieces of ropes around the flippers, head, or carapace were assumed to have been caught in this fishery. Finally, instances, where only large, severed portions of fresh carcasses were found, were categorized generally as interaction with vessels, likely a boat strike.

### 2.5. Data Analysis

Haplotype diversity (*h*) was estimated, according to Nei [62], using Arlequin v 3.11 [63]. Haplotype frequencies from our Uruguay sample set were compared using Pearson’s Chi-squared test with those published for stranded and bycaught leatherbacks in Argentina, including the southern portion of Rio de la Plata estuary [35], as well as coastal and oceanic areas off southern Brazil [33,34].

We performed a Bayesian mixed-stock analysis (MSA) to estimate the relative contributions of different nesting stocks to the foraging ground in the SWAO, using the program BAYES [64]. Based on a lack of differentiation between foraging grounds, also reported by Vargas et al. [34] for Brazil and Argentina, data were combined to represent a single SWAO foraging population (stock mix). The MSA was conducted using published mtDNA haplotype frequencies for eleven rookeries, sampled to date, in the Atlantic and Indian Oceans [33,34,35,54,65,66], for the rookery baseline (Table 1). Individual rookeries, whose haplotype frequencies were not significantly differentiated, were combined into the same MU (NWCG and SECG) [67], which in this study represents the seven genetic stock groupings described in Vargas et al. [34].

**Figure 2:**
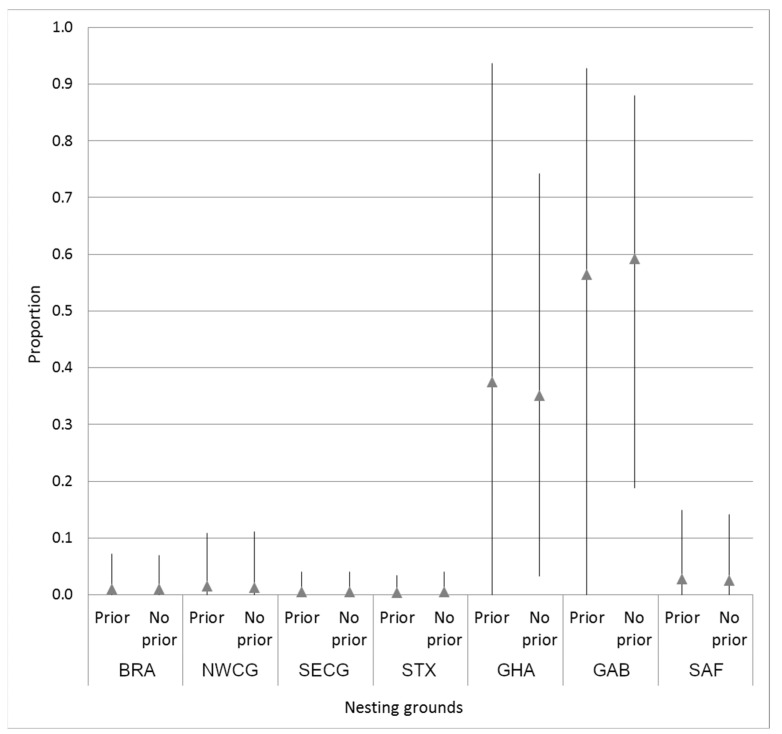
Mean estimated stock contributions from Atlantic leatherback rookeries to the combined UR, BA, and SBR regional SWAO foraging aggregation using priors (weighted by rookery size) and no priors (equal contributions). Credible intervals (95%) are indicated. Abbreviations correspond with Figure 1.

We conducted the MSA with uniform (flat) priors and weighted priors (Figure 2). Analysis with flat priors assumed each stock was equally likely to contribute individuals to the foraging populations. For the weighted prior MSA, the potential stock contributions were weighted relative to the nesting population size, as measured by the estimated number of females published for each rookery (Table 1). A total of 7 chains, each consisting of 15,000 Markov Chain Monte Carlo (MCMC) steps (chain length), based on 7 potential source stocks (RMUs) were run with different starting points. A burn-in of 10,000 steps was removed for each chain and the remaining 5000 steps (35,000 total MCMC samples) were used to calculate the posterior distribution. The Gelman and Rubin shrink factor diagnostic was computed to ensure that all chains had converged, as indicated by a shrink factor of less than 1.2 for each chain [63].

The log-likelihood ratio test (LRT, “anova” function in the “nlme” package [68]) was employed to test for differences in the average number of strandings per year and month. The statistical analysis was performed with RStudio 1-4.11 [69]. The Kernel density map was created with ArcGIS 10.3 [70].

## 3. Results

### 3.1. Genetic Diversity in Foraging Grounds

A total of 243 leatherbacks, either stranded (n = 223) or bycaught (n = 20) by artisanal fisheries, were recorded between 1997 and 2021 in Uruguay. A total of 59 leatherbacks, consisting of 55 strandings and 4 bycaught by artisanal fleets between 2002 and 2016, were sampled and successfully sequenced. The most common haplotype for UR was Dc1.1 (n = 43), followed by Dc1.3 (n = 8), Dc1.7 (n = 1), Dc3.1 (n = 2) and Dc9.1 (n = 1), and Dc13.1 (n = 4) (Table 1). Haplotypes Dc9.1 and Dc1.7 (GenBank accession #OQ621749) have not been identified at any of the Atlantic rookeries to date and were excluded in the MSA. The overall haplotype diversity was 0.452 +/−0.074.

Pearson’s Chi-squared test based on haplotype frequencies did not reveal significant differences between the UR, BA, and the SBR foraging areas (Chi-squared = 9.14, df = 14, *p*-value = 0.822).

The results of the MSA estimates show that the majority (mean 94%) of the leatherbacks foraging in the SWAO come from the West African rookeries (GB and GHA), with minimal contribution (<1%) coming from each of the other Atlantic rookeries (Figure 2). The flat and weighted MSA produced similar results, the credible intervals (CI) were wide in both, although these slightly narrowed with the flat priors. GAB contributed slightly more with an estimated mean of 58% (CI: 18–87% flat priors) than GHA, with an estimated mean of 36% (CI: 4–73% flat priors) (Figure 2; Supplementary Table S1).

### 3.2. Size Distribution, Sex, and State of Decomposition

**Figure 3:**
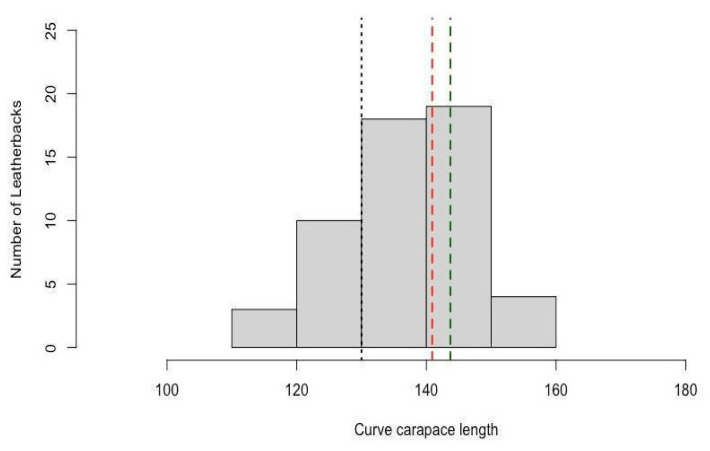
Curved carapace length (CCL) of leatherback turtles recorded in Uruguay (n = 98). Black dashed line indicates the minimum CCL of nesting females at 130 cm from the nearest nesting colony in Brazil (adapted from Thomé et al. [15]). Red dashed line indicates the mean size of females (n = 43) and the green dashed line the mean size of males (n = 21).

The sizes of the individuals ranged from 110.0–170.0 cm CCL, with a mean ± SD of 139.8 ± 11.6 cm (n = 98; Figure 3). A total of 34 specimens were classified as juveniles, 141 were classified as large juveniles/adults, and 68 were undetermined. Due to the high degree of decomposition of most specimens, only 64 individuals were able to be sexed, 43 as female (140.9 ± 8.4 cm) and 21 as male (143.7 ± 7.9 cm).

### 3.3. Spatiotemporal Distribution of the Strandings

**Figure 4:**
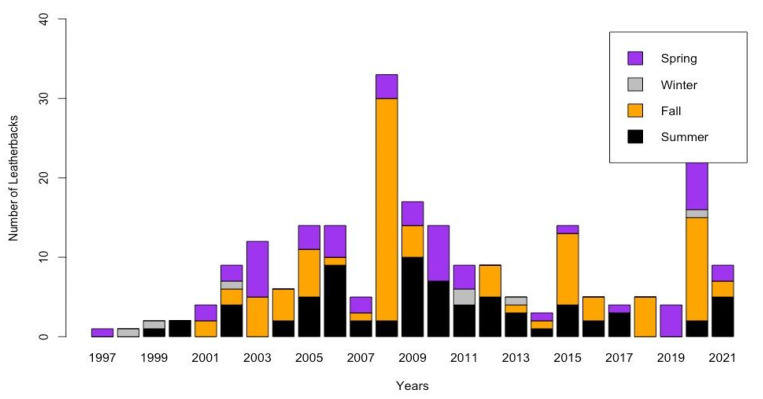
Number of stranded leatherbacks observed during the 25 years of the study (1997–2021), grouped by seasons (n = 223). Summer in black (n = 72), Fall in orange (n = 91), Winter in grey (n = 7), and Spring in purple (n = 53).

**Figure 5:**
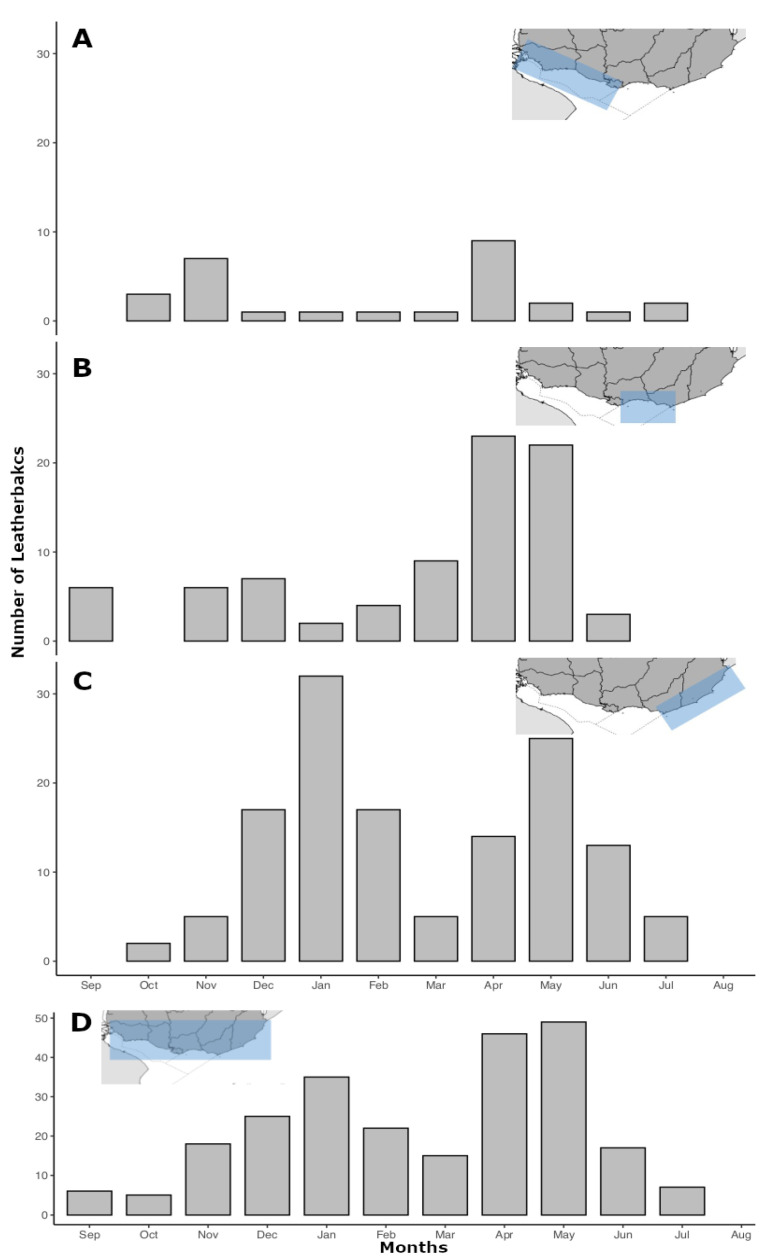
Number of stranded leatherbacks observed during the 25 years of the present study (1997–2021), per month, for each zone and all the Uruguayan coasts. (**A**) Inner estuarine zone (IEZ), (**B**) outer estuarine zone (OEZ), (**C**) oceanic zone (OZ), and (**D**) all the Uruguayan coasts (note the difference in the *y*-axis from the other panels). The blue polygon indicates the coastal area included in each panel.

The log-likelihood ratio test showed significant differences among the number of strandings, annually (LRT = 58.123, *p*-value < 0.0001) (Figure 4) and monthly (LRT = 63.116, *p*-value < 0.0001) (Figure 5). 

Leatherbacks were observed in all seasons, although only nine leatherbacks were ever documented in the Winter, while none were documented in August during the 25 years (Figure 4 and Figure 5). The highest percentages of strandings were recorded during the Fall (n = 91, 40.8%) and Summer (n = 72, 32.3%). The kernel density estimates of spatial distribution show that the highest number of strandings occurred in the oceanic zone (OZ; 60.9%, n = 136), followed by the outer estuarine (OEZ; 27.3%, n = 61), and the inner estuarine zones (IEZ; 11.6%, n = 26). Strandings occurred most often during the Fall (n = 91, 40.6% of the total) in the OZ (n = 42), OEZ (n = 38), and IEZ (n = 22), and during the Summer in the OZ (n = 73, 39.1% of the total) (Figure 5).

### 3.4. Cause of Strandings

**Figure 6:**
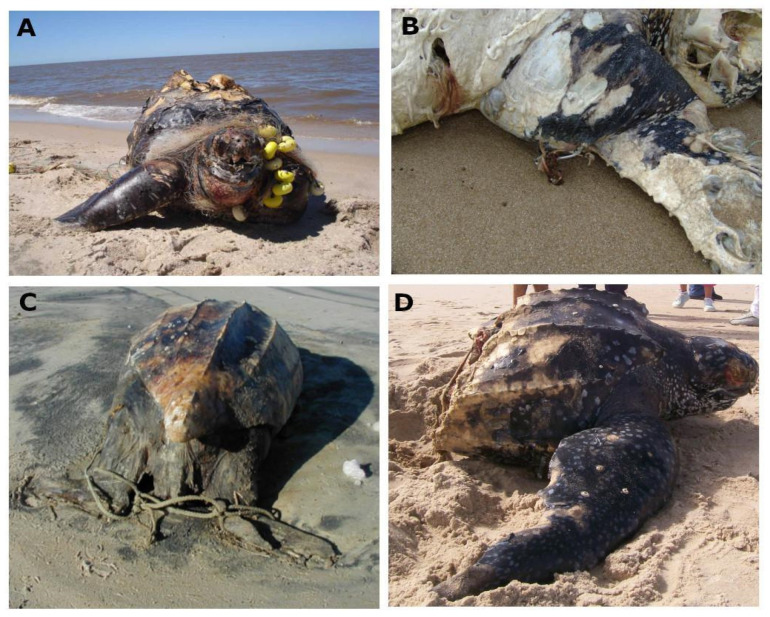
Stranded leatherback carcasses with evidence of human-caused interactions. (**A**) Artisanal set net entanglement, (**B**) longline fishing hook in the front flipper, (**C**) ropes probably used by fishers to move the turtle on an industrial fishing boat, and (**D**) leatherback cut in two pieces probably associated with boat collision. Photo credits: Karumbé.

Of the 223 stranded leatherbacks, 13 were found alive (5.8%), 45 were considered fresh dead (20.2%), 157 were in a moderate or advanced state of decomposition (70.4%), and 8 were not recorded. The live turtles received veterinary care and were measured, sexed, biopsied, and tagged with Inconel flipper tags prior to release. External examination of the carcasses did not reveal the cause of mortality in the majority of the cases (90.6%, n = 202). There was evidence of human interaction with the remaining incidents (9.4%, n = 21), mainly bycatch (n = 16), interaction with vessels (n = 4), and interaction with ghost nets (n = 1; Figure 6). When the cause of death was able to be determined, bycatch was higher than other threats and was determined to be a result of bycatch by different fisheries, as indicated by the presence of ropes, hooks, and other fishery gear. From the bycatch, we identified the interaction with artisanal fleets (set nets), in all zones mainly during the Fall and Spring.

## 4. Discussion

Our results expand the knowledge of this endangered species of marine turtle in the South Atlantic Ocean and reinforce the connectivity between the Southeast Atlantic rookeries and the SWAO foraging grounds to better inform conservation. The lack of differentiation found among the foraging areas of Uruguay (UR), Argentina (BA), and Brazil (SBR), indicates that the broader SWAO is part of the oceanic range for leatherbacks from the West African breeding populations, allowing updates to the delineation of the western boundary of the South Atlantic RMU [71]. The presence of large juveniles and adults in the study area occurred almost all year round with a higher presence during the austral Summer and Fall.

The MSA indicated that the majority (94%) of the leatherback turtles found in Uruguayan waters come from Ghana and Gabon in West Africa. These results are consistent with those obtained from Argentinian and Brazilian foraging grounds, demonstrating that leatherbacks of West African origin migrate to foraging areas in the Southwest Atlantic [33,34,35,37,54]. These results are consistent with a leatherback caught by an industrial trawler at the Rio de la Plata estuary in 2009, which had been previously tagged in Benin in 2004 (Karumbé, unpublished data).

The presence of rare, and unique “orphan” haplotypes in this study, namely Dc1.7, which have not been described for any nesting or foraging area, indicate that additional assessment of nesting populations is needed with larger sample sizes, in order to better characterize the genetic variation. Some African rookeries, notably Equatorial Guinea and Namibia, are missing from the baseline, and the sample size is inadequate from the existing rookery dataset to detect the rarer haplotypes. It should be noted that Dc9.1 has not been described to date for any nesting area in the Atlantic, yet has only been reported in the western and Indo Pacific [72]. It is unlikely that this indicates the movement of turtles from the Indo Pacific breeding areas to forage in the SWAO, but rather an artifact of incomplete baselines [73,74]. Furthermore, the presence of shared haplotypes among the rookeries contributes to the uncertainty of the mixed stock assignments. For instance, Dc3.1, which was present in SWAO foraging grounds, is present at rookeries throughout the Caribbean as well as Gabon in the east Atlantic, and potentially contributed to the estimate of some, albeit small, contributions from the Caribbean. All estimates have large CIs that span zero, indicating that they are probably just statistical “noise”. These results reinforce the need to use additional informative molecular markers, such as nuclear microsatellites, which have greater power to detect structure and assign origin than mtDNA for leatherbacks [54,75,76].

Given the extremely small size of the Brazilian rookery (between 6 and 92 nests per year [17]), the FG sample size would have to be much larger than in our study to be able to detect a contribution. Satellite-tracking of post-nesting females in Espírito Santo, Brazil, recently demonstrated the connectivity of these nesting grounds with both coastal and oceanic UR and BA foraging grounds [77]. These results once again highlight the importance of using additional more informative molecular markers to learn more about the connectivity among SWAO leatherback turtle populations.

The strandings documented in this study were formed of juveniles or adults, with a higher presence of adult-sized individuals. Given that the majority of the turtles in our study belong to the West African breeding populations, it will be more appropriate to use the sizes corresponding to adults for those rookeries in the future, as reported by Stewart et al. [78]. Gender was only determined in 28.7% of the turtles, and more females were identified (n = 43) than males (n = 21) (Figure 3). Our results also reflect the absence of small juveniles (<80 cm CCL) from the coastal waters of the South Atlantic, which are typically restricted to tropical waters between 5° N and 5° S [38]. Leatherback turtles are known to show behavioral plasticity as a result of changes in prey distribution and availability [79]. Furthermore, the swimming ability of juvenile turtles improves as they grow until they can swim against currents, as opposed to being passive drifters and, thus, as their dietary needs increase, larger turtles are able to direct their movements to seek out optimal foraging habitats over great distances [80,81], such as our study site. In Uruguayan waters, the leatherback’s main prey is the jellyfish (*Lychnorhiza lucerna*, Scyphozoa [82]). This jellyfish species has the same peak in strandings along the Uruguayan coast in the estuarine and oceanic zone during the warmer months between December and April (Personal communication V. Leoni) as the leatherback turtles. Furthermore, *L. lucerna* and *Chrysaora lactea* have been found in shallow coastal waters during Summer and Fall (December to early May) forming accumulations and mass occurrences between January and April [83,84], overlapping with the increased presence of the leatherbacks in shallow areas of Rio de la Plata and adjacent coastal waters [3,85,86].

The annual variability of leatherback strandings in our study area could also be associated with the large amplitude variations in continental runoff and winds at seasonal and interannual time scales that induce significant changes in the distribution of shelf water masses [87]. These variations in Río de la Plata discharge modulate the input and distribution of freshwater and nutrients, which lead to large changes in water mass properties over the neighboring continental shelf [88,89,90], affecting the marine food web and the presence of potential prey. The extremely high number of strandings we detected during April 2008 in the estuarine zone is likely associated with the unusual oceanographic conditions that occurred. Similar unusual peaks of strandings were also observed elsewhere in the SWAO region–for example, in October 2016 in Sao Paulo state [91] and November-December 2005 in Rio Grande do Sul State [42]; also in other regions, such as in La Guajira (Venezuela) during 2003 and 2013 [92]. These events reflect the need for more multidisciplinary studies to try to elucidate the main drivers of the interannual increase in the presence of leatherbacks at foraging grounds in the SWAO [3,41].

Determining the precise cause of stranding or death of turtles can be complicated due to factors such as carcass drift time and state of decomposition [93,94,95]. In our study, we were only able to determine the main cause of death in 9.4% of leatherback cases. Of these, the main cause was entanglement in fishing gear (Figure 6). The effects of bottom trawling fisheries were likely underrepresented due to the lack of external evidence, compared with the interaction with pelagic longlines and set nets. Our study area overlaps with areas of intense fisheries activities and boat traffic [96] due to the proximity of two of the main ports in the region, Montevideo (Uruguay) and Buenos Aires (Argentina). In this region, the Uruguayan and Argentinian fishing fleets carry out their activities throughout the year, yet the intensity increases during the austral Summer and Fall [43,97]. Previous studies indicated that coastal and oceanic waters in the region represent “hotspots” for interactions between leatherbacks and fisheries based on the overlap of the turtle occurrence and pelagic longline effort [14] and bottom trawler efforts, mainly in the estuary and maritime front of Rio de la Plata [98]. The higher effort of these fisheries [39,40,99] coincided with the months with a higher presence of leatherbacks in Uruguayan waters [3] and is the most plausible explanation for the patterns we observed of more strandings during the Summer and Fall in the oceanic and outer estuarine zone. Moreover, the other threats detected in our study, boat collision and marine debris ingestion, while less common, need to be taken into account. While we cannot say with certainty that the freshly severed carcasses were the result of boat strikes, there is an increasing presence of large cargo and passenger boat traffic in the Rio de La Plata estuary and the Uruguayan coastal waters, and our observations suggest the need to evaluate this threat further. These vessels use shipping lanes and anchorage areas that occupy ca. 1500 km, which the leatherbacks also inhabit [96]. Finally, the Rio de la Plata estuary and adjacent waters contain high amounts of plastic debris that accumulate along front lines, such as the turbidity front [100], which also serves to aggregate gelatinous zooplankton [101]. Leatherbacks may mistake marine debris for jellyfish prey or accidentally ingest plastic during feeding [102].

These threats have increased in recent decades and could continue to worsen and should, therefore, be addressed when developing conservation plans for the species in the South Atlantic RMUs.

## 5. Conclusions

Large juveniles and adult leatherbacks occur within the whole study area but are concentrated in the outer estuarine and oceanic zone. This, taken together with previous observations, indicate that this area represents a local high-use foraging area for leatherbacks [3,40].

Our study corroborates the importance of the Uruguayan waters for this threatened marine species, including the Rio de la Plata estuary, a coastal hotspot for large juveniles and adult leatherback turtles in the SWAO. This area is a prey-rich zone along the Atlantic Ocean, potentially supporting high densities of foraging leatherbacks, as documented by frequent records of turtles incidentally captured in artisanal and industrial fisheries and persistent stranding events of this species in the area.

Our results illustrate the need to coordinate conservation efforts across high-use areas and the migratory corridors that connect the territorial waters of the three countries in the SWAO (Brazil, Uruguay, and Argentina) and the distant countries of the West African breeding areas.

## Figures and Tables

**Table 1:** Haplotype frequencies for Atlantic and Indian Ocean leatherback rookeries and foraging grounds, based on 763 bp mtDNA sequences. Abbreviations correspond with Figure 1. NWCG includes ACR, FLA, and DR; SECG includes TRI, GUI, and FWI (adapted from Vargas et al. [33]). Sample size (N), haplotype diversity (*h*), and estimated average number of nesting females per year (NFpy) are shown.

	Nesting Areas											Foraging Areas			
		NWCG				SECG									
Haplotype	BRA	ACR	FLA	DR	STX	TRI	GUI	FWI	GHA	GAB	SAF	ES	SBR	BA	UR
Dc1.1	20	119	209	38	98	65	115	23	47	178	34	4	63	26	43
Dc1.3									11	12			8	4	8
Dc1.4									1		7		2	1	
Dc1.7															1
Dc2.1					21										
Dc3.1	15	10	10	4	4	11	28	5		5		3	2		2
Dc3.2		2				11	26	1							
Dc4.1									1	2		1	3		
Dc9.1													2		1
Dc13.1	1								1	35			14	2	4
Dc17.1			3												
Dc19.1		1													
DcA5							6								
DcC3							2								
N	36	132	222	42	123	87	177	29	61	232	41	8	94	33	59
*h*	0.531	0.183	0.112	0.1765	0.338	0.415	0.533	0.352	0.379	0.387	0.298	0.678	0.524	0.371	0.425
NFpy	50	2500	75	35	250	3000	5000	55	100	5000	50				

## Data Availability

Restrictions apply to the availability of these data. Data were available on request from the corresponding author with the permission of Karumbé.

## Supplementary Materials

The following supporting information can be downloaded at: https://www.mdpi.com/article/10.3390/ani13081285/s1, Table S1: Estimated mixed stock analysis (MSA) contributions from Atlantic leatherback rookeries to the SWAO foraging aggregation using flat priors (MSA1) and weighted rookery size priors (MSA2). The mean and upper and lower 95% credible intervals (CI) are shown. Abbreviations correspond with Figure 1. Figure S1: Detail of the study area and leatherback strandings recorded between 1997-2021 in Uruguay. The coastal waters could be divided in three regions: Inner Estuarine Zone (IEZ), Outer Estuarine Zone (OEZ) and Oceanic Zone (OZ) are shown as well as the Departments (CO: Colonia, SJ: San José, MO: Montevideo, CA: Canelones, MA: Maldonado; RO: Rocha). The kernel density of strandings is shown (dark blue indicates the zones with higher densities), as well as individual strandings (dots).
