# Supplementary material for: Natal Origin and Spatiotemporal Distribution of Leatherback Turtle (Dermochelys coriacea) Strandings at a Foraging Hotspot in Temperate Waters of the Southwest Atlantic Ocean
Source: Animals (Basel). 2023 Apr 8;13(8):1285. doi: 10.3390/ani13081285 (PMC10134985; doi:10.3390/ani13081285)
Supplement: Supplementary file 1 [file animals-13-01285-s001.zip › Supplementary_Table S1.pdf]

Supplementary Materials

Table S1: Estimated mixed stock analysis (MSA) contributions from Atlantic leatherback rookeries to the SWAO foraging aggregation using flat priors (MSA_1) and weighted rookery size priors (MSA_2). The mean and upper and lower 95% credible intervals (CI) are shown. Abbreviations correspond with Figure 1

Stock	MSA	Mean	Standard deviation	2.5%	Median	97.5%
BRA	MSA_1	0.0100	0.0202	0.0000	0.0007	0.0724
	MSA_2	0.0103	0.0200	0.0000	0.0007	0.0698
NWC	MSA_1	0.0146	0.0303	0.0000	0.0007	0.1081
	MSA_2	0.0132	0.0312	0.0000	0.0005	0.1113
SEC	MSA_1	0.0047	0.0113	0.0000	0.0002	0.0404
	MSA_2	0.0046	0.0120	0.0000	0.0002	0.0403
STX	MSA_1	0.0037	0.0097	0.0000	0.0001	0.0337
	MSA_2	0.0047	0.0120	0.0000	0.0002	0.0408
GHA	MSA_1	**0.3753**	0.2066	0.0001	0.3513	0.9369
	MSA_2	**0.3504**	0.1588	0.0331	0.3354	0.7419
GAB	MSA_1	**0.5642**	0.2064	0.0002	0.5872	0.9275
	MSA_2	**0.5916**	0.1636	0.1887	0.6046	0.8796
SAF	MSA_1	0.0277	0.0429	0.0000	0.0050	0.1486

	MSA_2	0.0252	0.0402	0.0000	0.0035	0.1414
