# Supplementary figures and images for: Natal Origin and Spatiotemporal Distribution of Leatherback Turtle (Dermochelys coriacea) Strandings at a Foraging Hotspot in Temperate Waters of the Southwest Atlantic Ocean
Source: Animals (Basel). 2023 Apr 8;13(8):1285. doi: 10.3390/ani13081285 (PMC10134985; doi:10.3390/ani13081285)

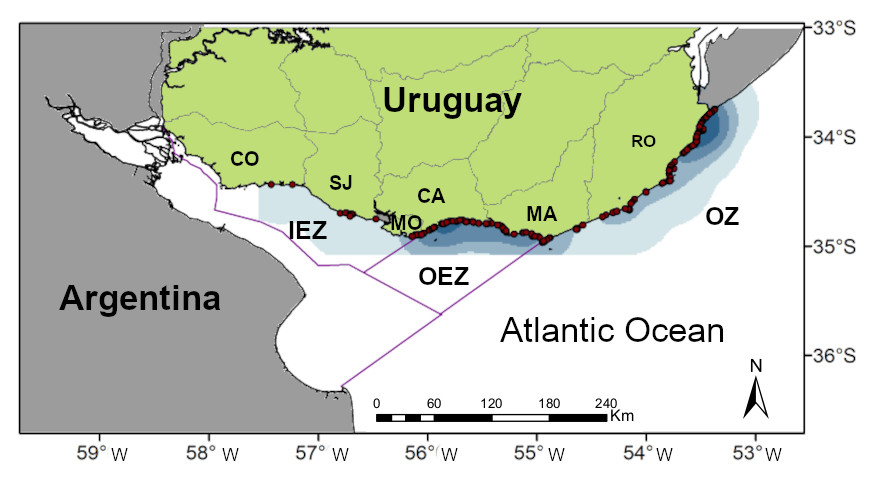

Supplement: Supplementary file 1 [file animals-13-01285-s001.zip › Supplementary_Figure S1.jpg]
